# Quantification of the Therapeutic Antibody Ocrelizumab in Mouse Brain Interstitial Fluid Using Cerebral Open Flow Microperfusion and Simultaneous Monitoring of the Blood–Brain Barrier Integrity

**DOI:** 10.3390/pharmaceutics15071880

**Published:** 2023-07-04

**Authors:** Thomas Altendorfer-Kroath, Joanna Hummer, Denise Kollmann, Beate Boulgaropoulos, Reingard Raml, Thomas Birngruber

**Affiliations:** Institute for Biomedical Research and Technologies (HEALTH), Joanneum Research Forschungsgesellschaft m.b.H, Neue Stiftingtalstrasse 2, 8010 Graz, Austria; joanna.hummer@joanneum.at (J.H.); denise.kollmann@joanneum.at (D.K.); beate.boulgaropoulos@joanneum.at (B.B.); reingard.raml@joanneum.at (R.R.); thomas.birngruber@joanneum.at (T.B.)

**Keywords:** cerebral open flow microperfusion, cOFM, blood–brain barrier, BBB, therapeutic antibody, absolute quantification, PK profile, pharmacokinetic, brain interstitial fluid

## Abstract

The increasing relevance of improved therapeutic monoclonal antibodies (mAbs) to treat neurodegenerative diseases has strengthened the need to reliably measure their brain pharmacokinetic (PK) profiles. The aim of this study was, therefore, to absolutely quantify the therapeutic antibody ocrelizumab (OCR) as a model antibody in mouse brain interstitial fluid (ISF), and to record its PK profile by using cerebral open flow microperfusion (cOFM). Further, to monitor the blood–brain barrier (BBB) integrity using an endogenous antibody with a similar molecular size as OCR. The study was conducted on 13 male mice. Direct and absolute OCR quantification was performed with cOFM in combination with zero flow rate, and subsequent bioanalysis of the obtained cerebral ISF samples. For PK profile recording, cerebral ISF samples were collected bi-hourly, and brain tissue and plasma were collected once at the end of the sampling period. The BBB integrity was monitored during the entire PK profile recording by using endogenous mouse immunoglobulin G1. We directly and absolutely quantified OCR and recorded its brain PK profile over 96 h. The BBB remained intact during the PK profile recording. The resulting data provide the basis for reliable PK assessment of therapeutic antibodies in the brain thus favoring the further development of therapeutic monoclonal antibodies.

## 1. Introduction

In recent years, therapeutic monoclonal antibodies (mAbs) specific for target antigens in humans have been developed as part of an emerging strategy to treat neurodegenerative diseases [1]. However, assessment of their pharmacokinetic (PK) remains challenging, as only about 0.1% to 1% of the therapeutic antibodies from the systemic circulation finally enter the brain due to the restrictive action of the blood–brain barrier (BBB) [2]. Several strategies have been developed to improve the brain penetration properties of such therapeutic antibodies and promising outcomes have increased the relevance of mAbs, thus strengthening the need to continuously and reliably measure the brain PK profiles of these therapeutics [3,4].

Since many targets of mAbs are located in the interstitial fluid (ISF) that is surrounding the brain cells, cerebral ISF is the compartment of choice to assess relevant antibody concentrations [5,6]. To date, there are only two technologies that can extract ISF from brain in a time-resolved manner, and enable subsequent quantification of large therapeutic molecules in the collected samples; these are microdialysis (MD) with a large molecular weight cut-off membrane [7,8,9] and open flow microperfusion (cOFM) [8,9,10]. MD and cOFM differ in the presence/absence of a membrane at the probe’s interface between perfusion fluid and brain ISF. The membrane-free cOFM probes allow prolonged sampling of diluted and otherwise unchanged ISF and the collected samples contain the whole molecule spectrum of the ISF [9]. This is in contrast to MD probes where the polymeric semi-permeable membrane only permits the sampling of substances up to a specific molecular weight, determined by the cut-off value of the membrane. Further, the MD membrane itself is susceptible to the adsorption of various ISF components, which might bias results and prevent prolonged sampling [7]. Both MD and cOFM can be applied in combination with different quantification protocols such as no net flux or zero flow rate [9,11,12,13] for direct and absolute quantification of substances inside the brain ISF.

The BBB integrity status is especially relevant during PK assessment as the measured antibody concentrations in cerebral ISF are only reliable when the cerebral ISF is collected with an intact BBB. cOFM probe insertion inevitably disrupts the BBB whose integrity, however, is restored after 14 days [14]. The restoration of BBB integrity is a progressive process in which the BBB is established earlier for larger than for smaller molecules. However, by now, the BBB integrity status during cOFM sampling has only been continuously investigated with sodium fluorescein as a marker molecule, which is about 400 times smaller than a therapeutic antibody. For large molecules of the size of a mAb (about 145 kDa) the BBB integrity status has never been assessed in a continuous manner, but only at a few well-defined time points during the cerebral ISF sampling [8,14].

Although antibody concentrations in brain ISF have been examined with cOFM before and a brain PK profile has been recorded for 48 h [9], accurate and time-resolved quantification of an antibody in the brain to record PK profiles requires cOFM sampling over an extended period of time with a reliable intact BBB for the respective therapeutic molecule during the entire sampling period.

The aim of this study was, therefore, to absolutely quantify the therapeutic antibody ocrelizumab (OCR) by performing continuous cOFM sampling in combination with the quantification protocol zero flow rate, and to record its PK profile in brain ISF for 96 h. Further, we aimed to monitor the integrity of the BBB in a time-resolved manner while recording the PK profile over the entire sampling duration using an endogenous tracer with similar molecular size and PK properties as OCR.

## 2. Materials and Methods

### 2.1. Animals

All work related to the animal study was conducted according to the European Community guidelines for animal care (European Communities Council Directive 2010/63/EU) and was approved by the Federal Ministry Republic of Austria for Education, Science and Research under the animal application number 2020-0.051.877. Male C57Bl/6 mice were purchased from Charles River Laboratories, Germany and delivered to the Division of Biomedical Research at the Medical University Graz. Prior to any experiments, the animals were allowed to acclimatize for at least seven days in their home cages. They had access to food and water ad libitum all the time. All animals were included in the represented data. 

The animals were distributed into three groups. In group one (cOFM sampling in combination with zero flow rate, five mice) and group two (PK profile recording and concomitant BBB integrity monitoring, five mice) one cOFM probe (Joanneum Research-Health, Graz, Austria) per animal was inserted and cOFM sampling was subsequently performed. The animals of group three (three mice) did not receive any cOFM probes and served as reference for plasma pharmacokinetics.

### 2.2. cOFM Probe Insertion and Sampling Preparation

One cOFM probe was inserted into the striatum region at the coordinates 0.5/1.8/−4 (AP/ML/DV in mm) in each animal of group one and group two. Insertion and post-surgical treatment were performed as has been described previously [15]. The mice were allowed to recover from surgery for 14 days post-probe insertion. On the day of sampling, the animals were connected to the sampling system under slight anesthesia induced by inhalation of isoflurane, 1% in 2 L/h O_2_. After the healing dummy had been replaced by a sampling insert the animals were connected to the tethering system (Raturn^®^, Bioanalytical Systems Inc., West Lafayette, IN, USA), and the cOFM probe was flushed with perfusate (Table 1) with a flow rate of 2 μL/min for 5 min. All perfusate compounds were purchased from Sigma-Aldrich, Wien, Austria.

### 2.3. Therapeutic Antibody

OCR, a mAb that binds to human CD20 antigen located on mature B cells, which have been associated with the development of Multiple Sclerosis, was applied as an injectable formulation (Ocrevus^®^, Roche, Basel, Switzerland) [16]. The OCR solution was prepared according to the manufacturer’s instructions. All animals were dosed with 20 mg/kg OCR intravenously via the tail vein. During dosing, the animals were under slight narcosis to reduce the stress of the animals and to avoid damage to the cOFM sampling system due to extensive animal movement. All animals were in normal condition and showed no signs of aberrant behavior post infusion.

### 2.4. cOFM Study

#### 2.4.1. cOFM Combined with Zero Flow Rate

cOFM sampling in combination with the quantification protocol zero flow rate was used to absolutely quantify OCR in brain ISF after intravenous administration of an OCR bolus of 20 mg/kg. The concentrations of OCR in brain ISF were calculated based on studies from Bungay et al. and Chen et al. [17,18].

To perform zero flow rate, each animal of group one was dosed six hours prior to connection to the tethering system. The cOFM probes were flushed with perfusate once and then the flow rate was set to 0.2 μL/min. After a run-in phase of one hour, the cOFM sampling started and cOFM samples were collected every four hours. The flow rates were increased stepwise after each sampling interval (from 0.2 μL/min to 0.3 μL/min, 0.5 μL/min, 1 μL/min, 1.5 μL/min, and 2 μL/min), and the mean OCR concentrations in the cerebral ISF samples were assessed for the different flow rates. The total sampling duration for the zero flow rate experiment was 24 h. After the experiment, the cOFM samples were stored in a refrigerated fraction collector (Bioanalytical Systems Inc., West Lafayette, IN, USA).

Blood samples were taken two minutes prior to dosing and at the end of the cOFM sampling procedure at t = 96 h. The blood samples were then centrifuged at 2000× *g* for 5 min at room temperature and the supernatant plasma was transferred into sample vials (0.2 mL PCR tubes, Corning, Amsterdam, The Netherlands). After having collected the final cOFM sample the animals were disconnected from the tethering system and euthanized with an intraperitoneal pentobarbital (300 mg/kg) injection, followed by transcardiac perfusion with phosphate-buffered solution (PBS) 1× and heparin.

Cerebral spinal fluid (CSF) was collected terminal from the cisterna magna at t = 96 h. The animals’ brains were removed from the skull and cut into 30 mg aliquots. All samples were stored at −80 °C until the performance of the analytical measurements.

#### 2.4.2. PK Profile Recording and BBB Integrity Monitoring

PK profile recording and concomitant BBB integrity monitoring were performed with the animals of group two.

PK profile recording: The cOFM probe was flushed with perfusate. Then the perfusate flow rate was reduced to 0.5 μL/min and after a run-in phase of one hour the cOFM sampling started. The first cOFM sample was used as the baseline sample (sampling time: −2 to 0 h, pre-dose) and dosing was performed immediately after having taken the baseline sample at t = 0. Then, cOFM samples were collected every 2 h and stored in a refrigerated fraction collector (Bioanalytical Systems Inc., West Lafayette, IN, USA) for 96 h.

Blood samples were collected in EDTA-coated sample vials (Sarstedt, Nuembrecht, Germany) two minutes prior to dosing and at the end of the cOFM sampling procedure at t = 96 h. Blood samples were centrifuged at 2000× *g* for five minutes at room temperature, and the supernatant plasma was transferred into sample vials. After termination of the cOFM sampling, the animals were disconnected from the tethering system and euthanized by pentobarbital (300 mg/kg; intravenously) injection, followed by transcardiac perfusion with PBS 1× and Heparin. 

CSF samples were collected terminal from the cisterna magna. Animals’ brains were removed from the skull and cut into 30 mg aliquots. All samples were stored at −80 °C until the analytical measurements were performed.

BBB integrity monitoring: The BBB integrity was monitored by assessing the mouse immunoglobulin G1 (mIgG1) concentrations in the cOFM samples. The obtained cOFM samples were divided into two aliquots, one for OCR analytics and a second one for mIgG1 analytics. Six aliquots per 24 h were used for mIgG1 analyses. 

### 2.5. Plasma PK

Antibody plasma PK was assessed with animals of group three. Blood samples were collected in coated vials at 2 min, 10 min, and 5 h, 24 h, 48 h, 72 h, and 96 h post dose. Blood samples were centrifuged at 2000× *g* for five minutes at room temperature and the supernatant plasma was transferred into sample vials. Plasma samples were stored at −80 °C until bioanalytical measurements.

### 2.6. Antibody Analysis

OCR was analyzed with a human NHP/Isotyping Kit (Kit# K15203D-1, Mesoscale discovery, Rockville, MD, USA) following the provider’s instructions with the following adaptation: The calibration standards were prepared with OCR in perfusate at concentrations from 3 to 205 ng/mL. The cOFM samples for OCR analysis were analyzed directly or diluted 1 + 1 in perfusate, the serum samples were diluted 1 + 12,000, the CSF samples were diluted 1 + 6, and tissue samples 1 + 3 in perfusate before analysis. The selectivity was evaluated with four blank mouse plasma samples diluted 1 + 1 in the perfusate. The signals in all these samples were below the limit of detection, and therefore no cross-reactivity with mouse immunoglobulins was observed. The accuracy and precision of the method were evaluated at 3 concentrations with 15 quality control (QC) samples per concentration analyzed in 3 different batches (5 QC samples per batch) at 8, 30, and 125 ng/mL. The accuracy was between 3% and 7%, and the precision was between 1% and 19%. The lower limit of quantification (LLOQ) of the method, determined with 8 samples in 3batches, amounted to 3 ng/mL. The accuracy and precision at the LLOQ were −1% and 4%, respectively.

The mIgG1 concentrations were measured from every second cOFM sample. They were analyzed with a Mouse Isotyping Panel 1 Assay (Kit# K15183B-1, Mesoscale discovery, Rockville, MD, USA) according to the provider’s instructions except that the calibration standards were prepared with mIgG1 in perfusate at concentrations from 0.024 to 100 ng/mL. The cOFM samples for mlgG1 analyzation were analyzed after 1 + 1 dilution in the perfusate. The serum samples were diluted 1 + 80,000 and tissue samples were diluted 1 + 3 and 1 + 64 in perfusate before analysis.

Brain biopsies were homogenized using a Bead Ruptor Elite (Omni, Kennesaw, GA, USA), and OCR and mIgG1 were extracted from the brain homogenate with 1 mL Tris-HCl buffer. The homogenized tissue in buffer was centrifuged with 10,000× *g* at 4 °C for 10 min, and the supernatant was transferred into sample vials for analysis. OCR and mIgG1 were analyzed as described above.

### 2.7. Data Processing and Modeling

Unless otherwise described, all data were presented as mean ± standard error (mean ± SEM) of the respective group. PK parameters were assessed by non-parametric methods using MS Excel 2016(Microsoft, Redmond, WA, USA). Drug exposure of the respective tissues was expressed as the area under the drug concentration versus the time curve from t = 0 h to t = 96 h (AUC_0h–96h_). AUC_0h–96h_ was assessed with the trapezoidal method. Values derived from brain homogenate were corrected for the ISF volume fraction (20%) of the total brain volume.

#### 2.7.1. Zero Flow Rate

A non-linear fit with the model function according to Bungay et al. [17] was applied to data from animal group one (Equation (1)) and extrapolation was done using OriginPro 8.5 (OriginLab, Northampton, MA, USA) and MS Excel.

(1) $$c_{cISF}=c_{0}\left(1-e^{\frac{-1}{Q\times R}}\right)$$

C_cISF_ and c_0_ are the mean OCR or mIgG1 concentrations in the cOFM samples at the respective flow rate and the absolute OCR concentration in brain ISF, respectively. Q depicts the respective flow rate and R describes the overall mass transfer resistance of the antibody. R was estimated based on the assumption that transmembrane resistance and intradialysate resistance are negligible with regard to the mass transfer resistance inside the brain tissue. Using the geometric parameters of the probe and the diffusion properties of IgG1-like antibodies in the brain tissue, the effective diffusion coefficient of a human IgG1-type antibody is 6.68 × 10^−8^ cm²/s [17,19]. Further, it has been assumed that the intra- and extracellular metabolism rate constants are negligible. The elimination rate constant k_e_ that is describing the efflux of OCR to the microvasculature was assessed by linear regression of the log concentration over a time curve.

#### 2.7.2. Relative Recovery (RR)

The in vivo relative recovery (RR) was calculated according to Equation (2).

(2) $$RR=\frac{c_{cISF}}{c_{0}}$$

The concentration values were corrected for RR (c_cISF,corr_) by

(3) $$c_{cISF,corr}=\frac{c_{cISF}}{RR}$$

## 3. Results

### 3.1. Zero Flow Rate and In Vivo Relative Recovery (RR) of the cOFM Probe

OCR in brain ISF was absolutely quantified by cOFM sampling combined with zero flow rate and subsequent bioanalysis of the collected samples. The mean OCR concentration in the cerebral ISF samples (c_cISF_) at each applied flow rate is presented in Table 2.

**Figure 1:**
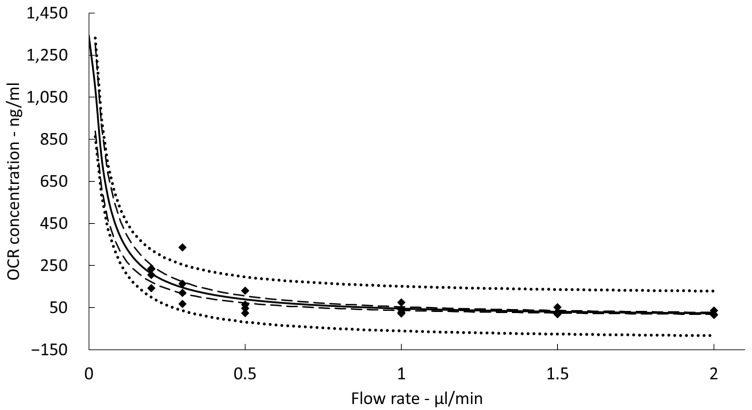
Ocrelizumab (OCR) concentrations from cerebral interstitial fluid (ISF) collected with cerebral open flow microperfusion (cOFM) at different flow rates (diamonds). Non-linear fit (solid line) with 95% confidence interval (dashed line) and 95% prediction interval (dotted line).

The overall mass transfer resistance (R) was calculated to be 29.17 min/mm. Extrapolation to a flow rate of zero of the OCR concentration versus flow rate curve yielded an absolute OCR concentration in brain ISF (c_0_) of 1342.9 ± 123.5 ng/mL (Figure 1).

The RR corresponding to the applied flow rate of 0.5 μL/min and the used cOFM probes were calculated to be 6.8%.

### 3.2. OCR PK Profile in Cerebral ISF

**Figure 2:**
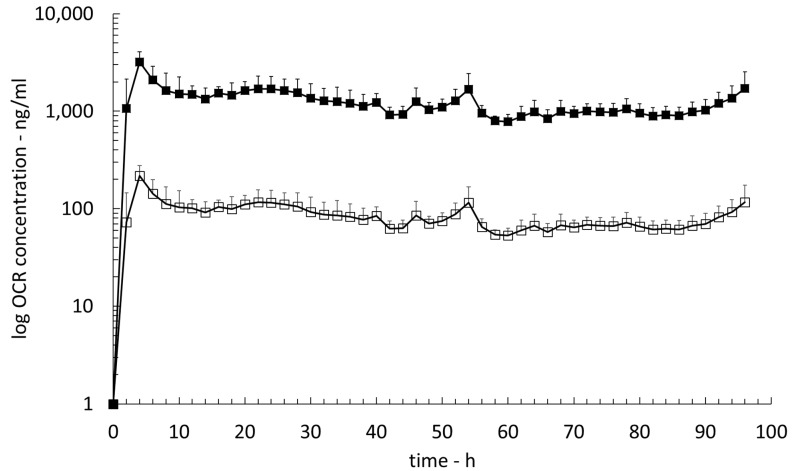
Pharmacokinetic (PK) profiles of OCR in cerebral ISF. Open squares: uncorrected OCR concentrations; solid squares: RR-corrected OCR concentrations.

The OCR concentration versus time profile (PK profile) was recorded for 96 h post dose (Figure 2). It did not show any significant variations throughout the entire sampling duration (SEM < 75 ng/mL throughout the sampling period). The maximum OCR concentration in cerebral ISF (C_max,cISF_) of 218.1 ± 50.5 ng/mL was reached at approximately four hours post dose. The area under the OCR concentration versus time curve from t = 0 h (dosing) to t = 96 h post dose (AUC_0h–96h_) was 8.0 μg.h/mL, and the half-life of OCR in brain ISF was estimated to be 112.8 h. The elimination rate constant (k_e_) from brain tissue was 0.006/h.

Cerebral ISF concentrations of OCR were corrected with the previously determined RR of 6.8%. The corrected maximum OCR concentration in cerebral ISF (C_max,cISF,corr_) was 3195.0 ± 869.9 ng/mL and the RR-corrected AUC_0h–96h_ (AUC_0h–96h,corr_) was calculated to 117.7 μg.h/mL.

### 3.3. OCR Concentrations in Plasma, Brain Tissue Homogenate, and Cerebrospinal Fluid (CSF) at t = 96 h

**Figure 3:**
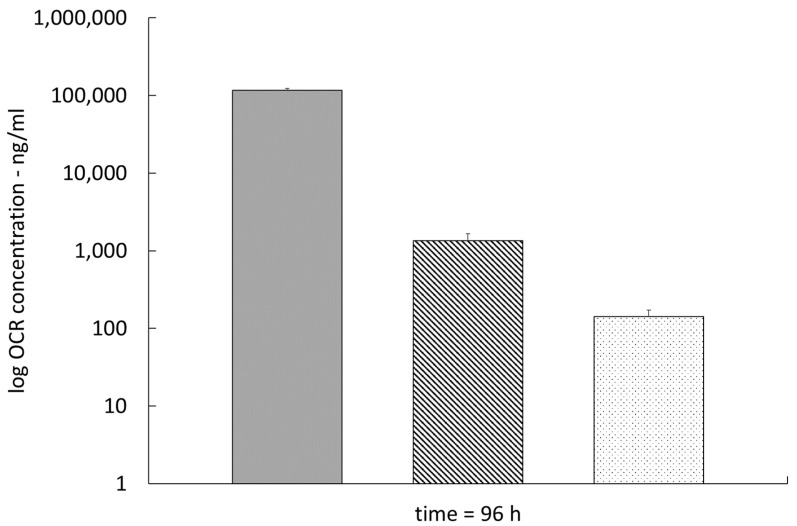
OCR concentrations in plasma (solid), brain tissue homogenate (hatched), and cerebrospinal fluid (CSF) (dotted) at t = 96 h (n = 5).

OCR concentrations in plasma, brain tissue homogenate, and CSF were 117,296.6 ± 5271.8 ng/mL, 1486.4 ± 343.7 ng/g, and 141.6 ± 30.1 ng/mL, respectively, at t = 96 h (Figure 3).

### 3.4. OCR PK Profile in Plasma

**Figure 4:**
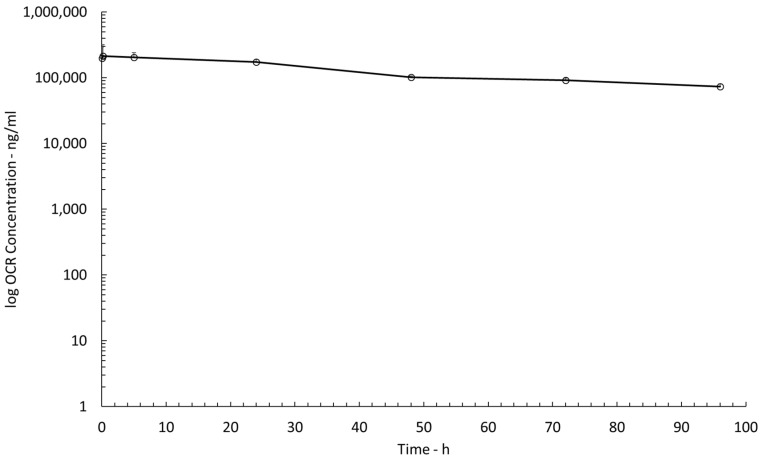
PK profiles of OCR in plasma (n = 3).

The resulting mean maximum plasma concentration of OCR (C_max, plasma_) was 213.2 ± 39.4 μg/mL (Figure 4). AUC_0h–96h_ in plasma was 18,150.4 μg.h/mL, yielding a volume of distribution for OCR of 3.4 L/kg. The overall elimination clearance was 0.037 L/kg.h. The elimination half-life of OCR in plasma was estimated to be 60.4 h. The ratio of the cOFM-derived AUC_0h–96h,RR_ to the plasma-based AUC _0h–96h_ was 0.01.

### 3.5. Continuous BBB Integrity Monitoring with Endogenous Mouse Immunoglobulin G1 (mIgG1)

**Figure 5:**
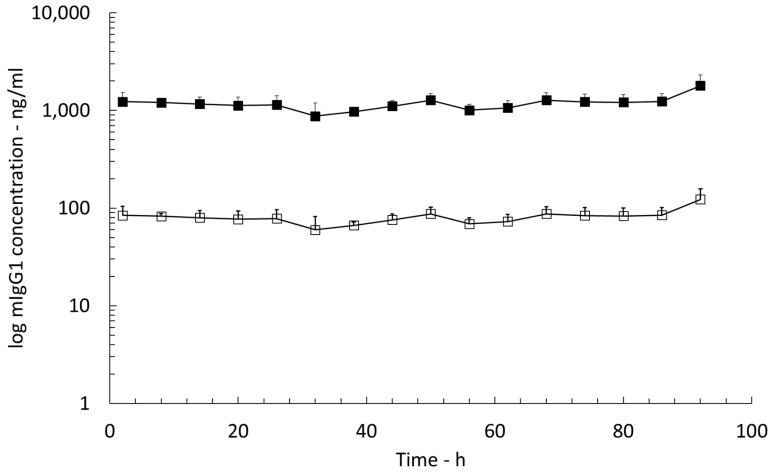
Mouse immunoglobulin G1 (mIgG1) ISF concentrations versus sampling time. Open circles: uncorrected ISF mIgG1 concentrations; solid circles: RR-corrected ISF mIgG1 concentrations, (n = 5).

**Figure 6:**
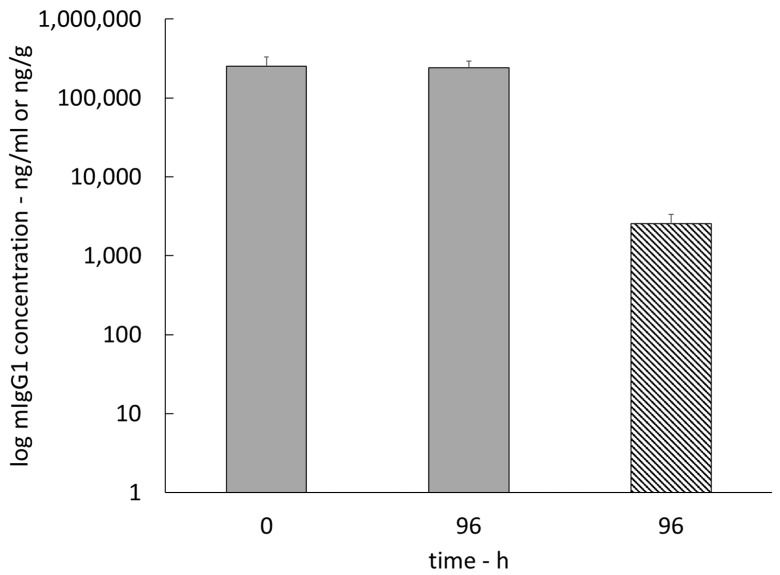
mIgG1 concentrations in plasma at t = 0 and 96 h (solid) and mIgG1 concentration in brain tissue homogenate at t = 96 h (hatched) (n = 5).

The concentration versus time curve of endogenous mIgG1 was recorded simultaneously with the PK profile of OCR in brain ISF for the entire PK profile recording of 96 h (Figure 5). The mean mIgG1 concentrations in cerebral ISF ranged from 59.7 ± 21.9 ng/mL to 122.5 ± 34.7 ng/mL, and the concentration versus time profile did not show significant variations throughout the entire sampling duration (SEM < 35 ng/mL from dosing to 96 h post dose). The mIgG1 concentrations in brain tissue homogenate were 2824.8 ± 848.9 ng/g at t = 96 h post dose (Figure 6), and the ratio of the mIgG1 concentrations in brain tissue homogenate to those in plasma was 0.01. The mIgG1 concentrations in plasma before and after OCR dosing were 251.2 ± 78.9 μg/mL and 242.6 ± 53.2 μg/mL, respectively (Figure 6).

Assuming that the mass transfer resistance in brain tissue of mIgG1 is similar to that of a human IgG1 therapeutic antibody we used the RR value of 6.8% to correct the mIgG1 concentrations. The RR-corrected concentrations of mIgG1 in brain ISF ranged from 874.7 ± 322.0 ng/mL to 1794.9 ± 509 ng/mL (Figure 5).

## 4. Discussion

We were able to directly and absolutely quantify the therapeutic antibody OCR in mouse brain ISF and to record the brain PK profile of OCR for 96 h by performing cOFM sampling, combined with the quantification protocol zero flow rate, and, subsequently, analyzing the cerebral ISF samples. Furthermore, our results demonstrated that the BBB remained intact for a large therapeutic antibody during the entire PK profile recording.

The continuous cOFM sampling to record the PK profile was stable for a duration of 96 h. The prolonged sampling duration allowed capturing a significant portion of the OCR’s PK profile, which is particularly important for such macromolecules that have plasma half-lives of days compared to small molecules with plasma half-lives in the order of hours. 

Up to now, there is one study that has demonstrated cOFM’s feasibility of continuously sampling antibodies in brain tissue, and that has recorded the PK profile of the therapeutic antibody in cerebral ISF [9]. In this previous study, trastuzumab has been used as a model antibody [9], which has about the same molecular size as the antibody we applied in our study. Both trastuzumab and OCR are humanized monoclonal IgG1s. However, in contrast to trastuzumab, which is licensed for the treatment of human epidermal growth factor receptor 2 positive breast cancer OCR is approved for the treatment of patients with relapsing or primary progressive form of multiple sclerosis [20,21,22,23]. OCR targets CD20^+^ B cells and depletes the number of these in circulation. Moreover, B cells have also been found in biopsies and autopsies of MS lesions indicating that OCR might have a target inside the brain parenchyma [24,25].

In contrast to our study, the study performed by Le Prieult et al. had a limited study duration of 48 h, and the absolute quantification procedure has been performed differently compared to the here presented study, particularly with regard to the cOFM-derived antibody concentrations [9]. Moreover, the BBB integrity status for the antibody during sampling remained unclear in this previous cOFM study [9].

In order to get reliable cOFM-derived antibody concentration values it is advisable to apply an RR-based correction factor to the measured concentration values. In the presented study here, we determined the in vivo RR of the respective cOFM probe by applying the same flow rates as in the PK study which we were then using for data correction. In general, the in vivo RR is affected by two factors: first, the flow rate of the perfusion fluid through the probe (that is determining the degree of dilution of the collected ISF); second, the mass transfer resistance of the antibody in the ISF [17,18].

Although the perfusate and the ISF can exchange freely through the open pores at the exchange area of the cOFM probe, the RR of a cOFM probe never reaches 100% and, therefore, cOFM samples always contain diluted cerebral ISF. This is because the perfusate, which is flowing continuously through the probe, is constantly replacing the volume at the interface between the probe and the ISF, and the time for volume exchange at this interface is usually too short to reach equilibrium between the perfusate and the ISF. Moreover, the molecules that had been removed from the tissue in the vicinity of the probe have to be replaced by molecules diffusing from the adjacent tissue to the probe. Diffusion in tissue is a more limiting factor for larger molecules than for smaller ones and becomes a main contributor to RR when sampling large molecules such as antibodies [26].

We assessed the in vivo RR based on the theoretical framework of Bungay et al. [17] that has initially been developed for MD. It relies on the assumption that the mass transfer resistance is composed of the mass transfer resistances in the semipermeable membrane, the perfusate, and the external medium [17,18]. As the cOFM probe lacks a membrane, the mass transfer resistance in a membrane can be set to zero in cOFM studies. Further, the mass transfer resistance of the antibody in the perfusate is negligibly small compared to that in the external medium (i.e., brain tissue), which is also supported by previous data showing that the diffusion coefficient of antibodies in the brain tissue is reduced by ten times compared to the free diffusion [17,19]. According to these considerations, the mass transfer resistance of a cOFM probe is determined exclusively by the resistance of the antibody in brain ISF.

In contrast to the in vivo RR, the in vitro RR of a probe mainly reflects the adsorption of the substance of interest to the probe material and, thus, it is of limited value when used as a measure to describe the in vivo sampling efficacy of a cOFM probe. Nevertheless, the in vitro RR can be of use to assess the degree of unspecific adsorption of compounds to the surface of the cOFM system. As a consequence, in vitro adsorption experiments are usually performed to optimize the perfusate composition, the setup, and the sampling regimen in order to reduce adsorption effects. Here, we corrected the measured OCR concentrations with the in vivo RR of the respective cOFM probe and, additionally, we performed in vitro tests prior to the cOFM sampling to assess adsorption of the antibodies (OCR and mIgG1) to cOFM system components to be used in the in vivo study. In the present study, we optimized the perfusate composition by adding 0.2% BSA to reduce the adsorption of the antibodies to the cOFM material. In agreement with the results from Custers et al. [8] we did not observe any adsorption of the antibodies to the cOFM system when using this optimized perfusate. 

For absolute quantification of an analyte in tissue after ISF sampling, different quantification protocols in combination with the sampling procedure are available [17,27,28,29,30,31]. The protocol of choice is determined by the properties of the analytes, e.g., molecular size, charge, diffusion, or PK properties. Because of the slow overall clearance and the slow diffusion of therapeutic antibodies in the ISF, zero flow rate was used as a quantification protocol in the cOFM study conducted here. Zero flow rate in combination with cOFM has also been applied previously [9]. However, in contrast to Le Prieult et al. [9], we calculated the resulting antibody concentrations based on the theoretical framework developed by Bungay et al. and Chen et al. [17,18].

Our PK data revealed a rather low elimination of OCR from brain ISF (k_e_ = 0.006/h) which is in agreement with results from other IgG monoclonal antibodies like trastuzumab [7,9,32]. Interestingly, the half-life of OCR in brain ISF exceeded the half-life of OCR in plasma. This has also been observed by Chang et al. [7] when investigating the humanized antibody trastuzumab. However, it is not clear yet if or why the antibodies accumulate in brain ISF. Different parameters affect the distribution and elimination of monoclonal antibodies such as the mode of action (e.g., antibody-dependent cellular cytotoxicity, complement-dependent cytotoxicity), the affinity of the antibody to different Fc-receptors (e.g., neonatal Fc-receptor or Fc-gamma receptor), the overall charge of the antibody molecule, the glycosylation pattern, or the route of administration (e.g., intravenous or subcutaneous) [33,34,35].

The onset of OCR in brain ISF was early (within the first four hours post dose) in our study, which is in good agreement with results obtained for the therapeutic antibody trastuzumab [7,9]. At the end of our cOFM PK study (at t = 96 h post dose), the in vivo RR-corrected OCR concentration in cerebral ISF matched the OCR concentration in the brain tissue, which is also well in line with recent cOFM data, but in contrast to results from previous MD studies [7,9]. Chang et al. [7] have applied the in vitro RR to correct the values derived from MD ISF samples. However, we assume that this could have led to an overestimation of the RR value, which resulted in an antibody concentration in ISF that was even higher than the total concentration of the antibody in the brain.

The OCR concentrations we found in CSF were lower than those derived from brain ISF which is consistent with results from Le Prieult et al. [9] who, like us, have collected CSF samples by puncturing the cisterna magna. In contrast to that, Chang et al. have collected the CSF from both lateral ventricle and cisterna magna samples with MD probes [7]. They then corrected the CSF concentrations from the dialysate of the MD probes with the in vitro RR, and the resulting antibody concentrations in the CSF from the lateral ventricle and cisterna magna were higher than the antibody concentrations in the brain tissue homogenate.

Again, it must be noted that correction by the in vitro RR might have led to an overestimation of the actual concentration values. The uncorrected cOFM values reported by Le Prieult et al. [9] were close to the concentration in brain tissue, but still below them [9], which is in line with our data. However, Le Prieult et al. [9] have used a lower sampling flow rate than we have (0.3 μL/min versus 0.5 μL/min). This lower flow rate leads to a higher in vivo RR when referring to Bungay et al. [17], which might explain the better correlation of the uncorrected cOFM concentration values to the brain homogenate concentration values.

The validity of brain ISF data strongly depends on the actual status of the BBB integrity for the substance of interest during data acquisition. In clinical practice, BBB integrity is routinely assessed with magnetic resonance imaging in combination with contrast agents (e.g., low-molecular-weight gadolinium-based contrast agents). However, because the BBB integrity is a dynamic and size-dependent state, monitoring it with a low-molecular-weight contrast agent is of limited value when assessing the PK of a high-molecular-weight molecule such as a therapeutic antibody in the brain. In this study, the BBB integrity status was continuously monitored throughout the entire PK profile recording with mIgG1 as a marker molecule for the first time. Endogenous mIgGs are well suited as marker substances when sampling therapeutic mAbs, as they are the most abundant antibodies in the blood of mice. Further, they have similar size and physical properties as mAbs as the majority of the therapeutic mAbs are based on human IgGs. Among the four IgG subtypes, IgG1 is the most prominent subtype with around 60% of total IgG concentration. The endogenous IgG1 concentration in plasma is generally stable and variations from the normal state of BBB integrity are indicated by elevated levels in brain tissue [36].

Until now, endogenous IgG has only been used for post hoc BBB integrity status assessment in immuno-histochemical studies, but it has never been collected from brain ISF by a cOFM probe to continuously assess BBB integrity [8]. In addition to indicating the integrity status of the BBB, endogenous IgG levels can also be used to indicate the functional status of the cOFM probe as abnormally low IgG levels in cOFM samples could reflect a malfunction of the cOFM probe.

The endogenous mIgG1 levels in our cOFM samples were stable throughout the sampling procedure, showing that the BBB integrity was not altered by the perfusion of the cOFM probe with the perfusate throughout the study and, further, that the cOFM probes were functioning properly. The former is in agreement with findings from Custers et al. [8], who have compared perfused versus non-perfused cOFM probes regarding BBB integrity by staining endogenous mIgG in brain slices. Their results have indicated that the BBB integrity is not affected by the perfusion of the cOFM probe 16 days after probe insertion. However, they have assessed the BBB integrity only at one single time point in the experiment and could therefore not draw any conclusions about the integrity status of the BBB during sampling [8].

In conclusion, cOFM enabled direct and absolute quantification of a therapeutic antibody (OCR) in brain ISF, and time-resolved, stable sampling allowed the recording of the antibody’s PK profile for a period of 96 h. Moreover, the BBB remained intact for a large molecule while recording OCR’s brain PK profile over this prolonged duration. This study provides evidence that cOFM can perform reliable PK assessment of therapeutic antibodies in brain ISF, delivering reliable and easily interpretable data for the development of therapeutic monoclonal antibodies and thereby promoting the development of therapeutic monoclonal antibodies to treat neurological diseases.

## Figures and Tables

**Table 1:** Composition of the perfusate.

Compound	Concentration [mg/L]
KCl	200
NaCl	8000
KH_2_PO_4_	200
Na_2_HPO_4_	1150
CaCl_2_	100.4
MgCl_2_	46.83
bovine serum albumin (BSA)	2000 (0.2%)

**Table 2:** Mean OCR concentrations ± SEM in cerebral ISF at the respective flow rates.

Flow Rate (μL/min)	Mean OCR Concentrations (ng/mL) ± SEM
0.2	203.8 ± 18.6
0.3	150.3 ± 44.6
0.5	64.5 ± 15.8
1.0	48.7 ± 10.2
1.5	34.0 ± 6.3
2.0	24.8 ± 4.6

## Data Availability

The datasets used and/or analyzed during the current study are available from the corresponding author upon reasonable request.
